# Evaluation of Conventional Radiography and an Electronic Apex Locator in Determining the Working Length in C-shaped Canals

**DOI:** 10.22037/iej.2017.12

**Published:** 2017

**Authors:** Hamid Jafarzadeh, Masoud Beyrami, Maryam Forghani

**Affiliations:** a *Dental Research Center and Department of Endodontics, Dental School, Mashhad University of Medical Sciences, Mashhad, Iran; *; b * Private Practice, Tehran, Iran; *; c * Dental Materials Research Center and Department of Endodontics, Dental School, Mashhad University of Medical Sciences, Mashhad, Iran*

**Keywords:** C-shaped Canals, Electronic Apex Locator, Radiography, Working Length

## Abstract

**Introduction:**

The purpose of this *in vitro* study was to compare the accuracy of working length determination using the apex locator versus conventional radiography in C-shaped canals.

**Methods and Materials:**

After confirming the actual C-shaped anatomy using cone-beam computed tomography (CBCT), 22 extracted C-shaped mandibular second molars were selected and decoronated at the cemento-enamel junction. The actual working length of these canals were determined by inserting a #15 K-file until the tip could be seen through the apical foramen and the working length was established by subtracting 0.5 mm from this length. The working length was also determined using conventional analog radiography and electronic apex locator (EAL) that were both compared with the actual working length. The data was statistically analyzed using paired t-test and marginal homogeneity test.

**Results:**

There was no significant differences between the working length obtained with apex locator and that achieved through conventional radiography in terms of measuring the mesiolingual and distal canals (*P*>0.05); while, significant differences were observed in measurements of the mesiobuccal canals (*P*=0.036). Within ±0.5 mm of tolerance margin there was no significant difference between EAL and conventional radiography.

**Conclusion:**

The apex locator was more accurate in determination of the working length of C-shaped canals compared with the conventional radiography.

## Introduction

Accurate determination of working length is one of the most important steps for successful root canal therapy. The apical constriction, which is the ideal apical end point for instrumentation in a tooth with complete root formation [1], is located within 0.5-1 mm short of the major apical foramen [2]. The apical foramen is frequently located, in an eccentric fashion, well away from the anatomic or the radiographic apex [3]. This makes it difficult to localize the apical constriction using the radiographic length determination technique [4].

A “C-shaped canal”, which was first termed by Cooke and Cox in 1979 [5], results from the fusion of the mesial and distal roots either on its buccal or lingual aspects. In general, the C-shaped canal has a single ribbon-shaped orifice with a 180^º^< arc [6]. However, below the orifice level, the root canal system can present a wide range of anatomical variations; not always continuously C-shaped from orifice to the apical foramen. Cheung *et al.* [7] investigated the apical anatomy of mandibular second molars with C-shaped canal system using micro-computerized tomography (µCT) and stereomicroscopy. According to their study, most of the teeth had two or three root canals, with almost 30% possessing either two or three apical foramina. The prevalence of accessory foramina was also about 48%. The complexities associated with the anatomy of the area near the apical region suggest that determination of working length, cleaning, shaping, and filling of the C-shaped canal system would be a great challenge [8, 9].

Many studies have evaluated the accuracy of working length determination using electronic apex locators (EALs) [10-12]. It is suggested that working length determination by using EAL may perform better than radiography alone [10], and reduce the frequency of over-instrumentation [11, 12].

However, a recent systematic review put an emphasis on the precision of electronic working length measurement which depends on the device used [13]. The Root ZX apex locator (J. Morita corp., Tokyo, Japan), which has been the object of numerous *in vitro* and *in vivo* studies, ensures a high level of reliability in locating the apical foramen [14] and the apical constriction [15]. 

To the top of our knowledge, there is no information in current literature regarding the accuracy of working length determination using radiographic or electronic methods in treatment of C-shaped canals. The purpose of this *in vitro* study, therefore, was to determine if there is any difference in the accuracy level of EAL and radiographic method in determining the working length in C-shaped canals.

## Materials and Methods

The research protocol was approved by the Vice Chancellor of Research of Mashhad University of Medical Sciences (Grant No.: 920409). The actual C-shaped anatomy of 50 extracted human mandibular second molars with fused buccal or lingual roots, was evaluated using cone-beam computed tomography (CBCT) and only in 22 teeth C-shaped anatomy was confirmed. C-shaped canals were included in this study that had an arc connecting mesiobuccal and distal canals and a distinct single mesiolingual canal. Selected teeth were kept in 2.5% sodium hypochlorite for 2 h and then stored in 0.9% saline solution. The teeth were sectioned at the cemento-enamel junction using a diamond disc to provide unrestricted access to the canal space and to produce a reliable occlusal landmark for length measurement.

The patency of the apical foramen was then confirmed using #10 K-file (Maillefer Dentsply, Ballaigues Switzerland). Pulp tissue was removed partially and irrigation was performed with 3 mL of 5.25% sodium hypochlorite, followed by 3 mL saline, in order to remove debris from the canal space.

The actual working length (AWL) was measured by inserting a #15 K-file with double silicone stop into the root canal until the file tip was just visible through the apical foramen under 40× magnification using the Microscope (AM413FIT Dino-Lite Pro, Electronics corporation, Taipei, Taiwan). The silicone stopper was also adjusted to occlusal reference plane to this length. The distance between the file tip and the stop was measured with a digital caliper (Mitutoyo, Tokyo, Japan). Eventually, 0.5 mm was deducted from this length to obtain AWL.

For radiographic determination of working length (RWL), each tooth was mounted on a wax plate with dimensions corresponding to the size #2 of an intra-oral periapical film (Eastman Kodak Co., Rochester, NY, USA). All radiographies were taken using x-ray generator Flash Dent (Villa Sistemi Medicali, Buccinasco, Italy), which was set at 70 kVp, 8 mA and exposed for 0.4 sec, with the distance from the source to the film set at 20 cm. The preoperative radiography was taken employing the parallel technique. A #15 K-file was inserted into the mesiobuccal and mesiolingual canals with minimal force to a distance 1 mm less than the tooth length. In the same manner, #20 K-file was used for the distal canal. Subsequently, the working length radiography was taken with the measuring files in place at a mesial angle of approximately 20^°^, and the working length was corrected where required. When the tip of the files appeared radiographically not to be within the 0.5 mm range of the desired position, correction was made. A second radiography was taken with the corrected files and the final measurement was recorded as the radiographic working length. Measurement of the RWL was performed from the silicone stop to the file tip by two calibrated examiners with the aid of the digital caliper after film placement on a negatoscope.

As for the electronic length measurement (EWL), the teeth were placed in a conducting medium of alginate (Chromogel alginate, Marlic Medical Ind. Co, Tehran, Iran). The metal lip clip was attached to the alginate, and the same file that had been used for the radiographic length determination was attached to the file holder. The measuring file was advanced within the root canal until reaching the display level of “0.0” on EAL monitor. The file was then withdrawn until the display level of 0.5 was achieved. According to the manufacturer, the 0.5 millimeter reading when using the Root ZX mini indicates that the tip of the file is in/very near the apical constriction, and so it was at this point when the distance from the silicone stop to the file tip was recorded. Each root canal measurement was performed twice by the same operator.

Differences between the EWL, RWL and AWL were calculated. Positive and negative values indicated that the tip of the file must have been detected longer or shorter of the AWL, respectively.

***Statistical analysis***

Paired t-test was used to analyze the significance of the mean difference between results from the apex locator and those of the radiographic technique. The proportion of the measurements within a tolerance of ±0.05 mm was also calculated. Statistical analysis was performed based on marginal homogeneity test, with statistical significance set at 0.05.

## Results

The mean difference and standard deviation between the values obtained from each measurement technique and the AWL are shown in Table 1. A statistically significant difference was detected between the two methods for case of mesiobuccal canals (*P*=0.036). However, with respect to the mesiolingual and distal canals the difference was not significant (*P*=0.176 and *P*=0.485, respectively). Table 2 presents the percentage of measurements which fell short, long, or within ±0.5 mm of tolerance margin. In 76.4% of all canals (42 of 55), the tip of the file, measured radiographically, was within the range of ±0.5 mm. About 89.1% of EWL measurements were within this range (49 of 55). There was no significant difference in terms of precision between EWL and RWL (*P*<0.05). 

## Discussion

The purpose of this study was to evaluate the accuracy of EWL and RWL determination in C-shaped canals. The results showed that the apex locator was more accurate in determination of the working length in C-shaped canals compared to the conventional radiography.

Second molars were chosen for this study because a high prevalence of C-shaped canals has been reported in these of teeth [16]. In the present study, the AWL was established to be 0.5 mm coronal to the major foramen, as suggested in previous studies [17-19]. A digital caliper accurate to 0.01 mm was used for measuring the recorded distances to reduce possible measurement errors. 

Different embedding media have been used as conductive environments such as agar, gelatin, alginate, saline, and flower sponge soaked in saline, in the laboratory tests on apex locators [20]. Alginate showed good results in EWL measurement and simulated the periodontal ligament with its colloidal consistency [20, 21]. In addition to these, the ease of preparation and the low cost make it a good choice for use *in vitro* studies 

According to several authors, the depth of apical constriction is not uniform, and the apical anatomy varies from apex to apex [22-24]. However, Tsesis *et al.* [13] in their systematic review and meta-analysis mentioned that Root ZX has a high precision in determining the distance between the file tip and the apical constriction. According to the manufacturer, which claims the Root ZX mini apex locator can actually detect the apical constriction, and considering that the working length in general, is the length measured to the apical constriction, the present study used the 0.5 reading as the measuring point.

In this study a #15 K-file was used to determine the RWL; however in distal canals #20 K-file was choose because it was 

small enough to negotiate the total length of the canal but large enough not to be loose in the canal.

In the present study, the ±0.5 mm range from AWL was used as a useful indicator for clinical acceptability [25]. In 76.4% of all canals, the tip of the file, measured radiographically, was closer to RWL than 0.5 mm. 89.1% of EWL measurements were within the range of ±0.5mm. According to earlier studies, the Root ZX showed an accuracy of 90% to within 0.5 mm of the apical foramen or the cemento-dentin junction [26, 27], while the conventional radiographic method proved accurate in less than 90% of the times. 

Based on our results, using the radiographic method meant that 20% of canals were overestimated. However, when applying the electronic method, the WL overestimation stood only at 7.3%. This difference may be important clinically. Instrumentation beyond the apical foramen should be avoided, since it may cause postoperative pain and sensitivity, and obturation beyond the radiographic apex reduces the success rate of root canal therapy [28]. El Ayouti *et al.* [12] showed that application of the Root ZX resulted in reduced incidence of working length overestimation, compared with conventional radiography. Previous studies reported 2.56% [29] and 7.9% [30] overestimated measurements when using the Root ZX, which are in agreement with our results. 

The mean distance between the RWL and AWL was 0.376 mm. This distance in electronic method was 0.261 mm. The difference between the results of the two methods was statistically significant. When the canals were evaluated separately, it was found that the difference in measurement length was only significant in mesiobuccal canals (the mean distance for RWL and EWL was 0.253 mm and 0.403 mm, respectively). It should be mentioned that in C-shaped mandibular molars, the mesiolingual canal is separate, while the mesiobuccal canal swings back and merges with distal canal [6]. However, high percentage of multiple foramina in C-shaped teeth [7] revealed that the mesiobuccal canals may have separate portals of exit that are located well away from the radiographic apex. This could mean higher accuracy of apex locators in determination of working length of such canals. According to our results, using the mini Root ZX can reduce the risk of overestimating the root canal length in C-shaped canals. 

**Table 1:** Mean (SD) between the each measurement technique and real length

	**Apex locator**	**Radiography**
**MB**	0.253 (0.230)	0.403 (0.287)
**ML**	0.253 (0.230)	0.386 (0.275)
**D**	0.261 (0.341)	0.347 (0.402)
**Total**	0.261 (0.274)	0.376 (0.331)

**Table 2:** Numbers and percentages of measurements falling short, long, or within ± 0.5 mm tolerance margin

	**Apex locator (%)**			**Radiography (%)**		
	<0.05	±0.05	>0.05	<0.05	±0.05	>0.05
**MB**	4.5	81.8	4.5	5.0	75	20
**ML**	0.0	92.3	7.7	0.0	76.9	23.1
**D**	4.5	86.4	9.1	4.5	77.3	18.2
**Total**	7.3	89.1	3.6	20	76.4	3.6

## Conclusion

The apex locator was more accurate in determination of the working length in C-shaped canals compared with the conventional radiography. The Root ZX mini apex locator can reduce the risk of overestimation of the root canal length in these canals. However, the results of this *in vitro* study need to be verified by clinical studies.
